# Interleukin 2-Based Fusion Proteins for the Treatment of Cancer

**DOI:** 10.1155/2021/7855808

**Published:** 2021-11-08

**Authors:** Alana MacDonald, T.-C. Wu, Chien-Fu Hung

**Affiliations:** ^1^Department of Pathology, Johns Hopkins University, Baltimore, MD 21287, USA; ^2^Department of Oncology, Johns Hopkins University, Baltimore, MD 21287, USA; ^3^Department of Obstetrics and Gynecology, Johns Hopkins University, Baltimore, MD 21287, USA; ^4^Department of Molecular Microbiology and Immunology, Johns Hopkins University, Baltimore, MD 21287, USA

## Abstract

Interleukin 2 (IL-2) plays a fundamental role in both immune activation and tolerance and has revolutionized the field of cancer immunotherapy since its discovery. The ability of IL-2 to mediate tumor regression in preclinical and clinical settings led to FDA approval for its use in the treatment of metastatic renal cell carcinoma and metastatic melanoma in the 1990s. Although modest success is observed in the clinic, cancer patients receiving IL-2 therapy experience a wide array of side effects ranging from flu-like symptoms to life-threatening conditions such as vascular leak syndrome. Over the past three decades, efforts have focused on circumventing IL-2-related toxicities by engineering methods to localize IL-2 to the tumor or secondary lymphoid tissue, preferentially activate CD8^+^ T cells and NK cells, and alter pharmacokinetic properties to increase bioavailability. This review summarizes the various IL-2-based strategies that have emerged, with a focus on chimeric fusion methods.

## 1. IL-2 Biology

Since its discovery in 1976 by Morgan and colleagues, IL-2 biology has been well characterized [1, 2]. IL-2 was initially described as a T cell growth factor key for maintaining long-term cultures of T cells [1]. IL-2 is largely secreted by antigen-activated T cells and functions physiologically to aid proliferation and differentiation shortly after T cell receptor (TCR) stimulation [2, 3]. IL-2 signaling is a key contributor to downstream T cell fate through activation of different transcription factor programs [4]. T cells that are exposed to strong IL-2 signaling express BLIMP-1 and become terminally differentiated short-lived effector cells. Conversely, T cells that experience weak IL-2 signaling tend to favor development into a long-lived memory cell phenotype driven by the transcription factor BCL-6 [4]. This reinforces the belief that the environment in which a T cell receives stimulation signals is incredibly important for driving the immune response.

The IL-2 receptor (IL-2R) consists of three subunits, IL-2R*α* (CD25), IL-2R*β* (CD122), and IL-2R*γ* (*γ*_c_; CD132). The IL-2R is not constitutively expressed as a trimer, and three different iterations can exist on immune cells [3]. The intermediate affinity receptor consisting of IL-2R*βγ* (K_D_ = 10^−9^) is expressed by naïve CD4^+^ and CD8^+^ T cells, memory T cells, and natural killer (NK) cells [2]. The low-affinity receptor, consisting of IL-2R*α* (K_D_ = 10^−8^), is expressed transiently in T cells and is induced through the integration of T cell receptor and costimulation signals [5]. When IL-2R*α* binds IL-2, it causes a conformational change that can accommodate the association with IL-2R*β* and, finally, *γ*_c_ to form the high-affinity trimeric receptor, IL-2R*αβγ* (K_D_ = 10^−11^) [6].

IL-2R*α* lacks a long cytoplasmic tail, requiring association with IL-2R*βγ* to induce signal transduction [7, 8]. In contrast, IL-2R*β* and *γ*_c_ have long cytoplasmic tails that contain docking sites for Jak1 and Jak3, respectively, that phosphorylate signal transducer and activator of transcription 5A and 5B (STAT5) upon activation. Phosphorylated STAT5 molecules can dimerize or tetramerize, leading to transcription of several key T cell genes, including IL-2 itself. IL-2 signals through other central pathways, including Ras/MapK and PI3K/Akt, that are critical for survival, proliferation, and differentiation during an immune response [4, 5].

## 2. IL-2 in Cancer Immunotherapy

### 2.1. Origins of IL-2 Therapy

IL-2 became one of the first candidates for cancer immunotherapy following its discovery as a key factor for T cell functioning [1]. CD8^+^ T cells are an integral part of the adaptive immune system and are important for recognizing and removing virally infected and malignant cells. As such, the ability to generate a strong, antigen-specific CD8^+^ T cell response is critical for killing tumor cells. Analysis of human tumor immune infiltrate suggests that the presence of CD8^+^ T cells in the tumor microenvironment (TME) correlates with improved disease outcomes and has positioned CD8^+^ T cells as a central mainstay of immunotherapeutic strategies [9]. Preclinical studies with IL-2 therapy were successful, leading to clinical trials starting in 1985 [10]. In 270 patients who received high-dose IL-2 therapy for the treatment of metastatic melanoma, the overall response rate was 16%, with 17/270 complete responses and 26/270 partial responses [11]. Similarly, in 255 patients with metastatic renal cell carcinoma (RCC), there was a 14% overall response rate, with 12/255 complete responders and 24/255 partial responders [12]. As a result, high-dose IL-2 therapy received FDA approval in 1992 for metastatic RCC and 1998 for metastatic melanoma [10]. Although clinical response rates were modest, they laid the groundwork for the development of IL-2-based immunotherapies for cancer treatment.

Along with IL-2, other cytokines that are important regulators of adaptive immunity have been developed for immunotherapeutic applications [13]. Currently, type I interferon, IFN*α*, is the only other FDA-approved cytokine for the treatment of cancer, specifically hematological malignancies, hairy cell leukemia, Kaposi sarcomas, chronic myelogenous leukemia, renal cell cancer, and high-risk state II and III melanoma [13]. In addition to IFN*α*, cytokines such as IL-12, IL-15, IL-21, and IFN*β* are currently undergoing clinical testing [13, 14]. Much like IL-2 therapy, these cytokines are limited by half-life, toxicities, and low efficacy which has warranted the development of next-generation versions [15].

### 2.2. Shortcomings of Traditional IL-2 Therapy

It has become abundantly clear that the side effects and toxicities related to IL-2 therapy have limited the clinical applications [16, 17]. Clinical administration of IL-2 is highly regulated and requires specialized care centers that are prepared to manage the extreme range of toxicities, as well as the frequent dosing regimen [16]. The most prominent side effect of high-dose IL-2 therapy is vascular leak syndrome (VLS), characterized by increased endothelial cell permeability and the movement of fluid into the extravascular spaces. VLS has several consequences that are potentially life-threatening to patients, including hypotension, pulmonary edema, liver cell damage, elevated levels of liver enzymes in the serum, and decreased blood oxygen saturation.

The mechanism for how VLS occurs in patients receiving IL-2 therapy has been sought after for many years. Early research suggested that VLS was a consequence of IL-2 acting indirectly on endothelial cells. Secretion of proinflammatory cytokines, such as TNF*α*, IL-1*β*, and lymphotoxin from IL-2 activated effector cells, presumably CD4^+^ and CD8^+^ T cells and NK cells, resulted in vasodilation of endothelial cells [18]. In contrast, Krieg et al. demonstrated that CD31^+^ endothelial cells express low to intermediate levels of the high-affinity trimeric IL-2R and IL-2 can act directly on those cells to induce pSTAT5 [19]. Studies using mixed bone marrow chimeras showed that wild-type (WT) bone marrow cells transferred to irradiated CD25^−/−^ hosts resulted in no observable pulmonary edema. However, pulmonary edema was prominent when CD25^−/−^ bone marrow cells transferred to irradiated WT were treated with IL-2, underscoring a role for nonimmune cell IL-2R expression in the onset of IL-2-induced toxicities. Additional factors such as angiopoietin 2 in the serum and endothelial nitric oxide synthase (NOS) (but not inducible NOS) have been associated with IL-2-induced VLS (17–19).

Additionally, studies have implicated innate and adaptive immunity in the onset of IL-2-induced VLS. Antibody depletion of NK cells, but not T cells, which diminished IL-2-induced VLS did not impact treatment efficacy, emphasizing the role of the innate immune system [20]. This hypothesis was supported by Assier et al. who utilized genetic knockout mice to reveal what immune cells were responsible [21]. Recombination-activating gene knockout mice (Rag^−/−^), which lack B and T cells, were still able to develop VLS, but Rag^−/−^ IL-15^−/−^ double knockout mice (no B, T, or NK cells) were resistant to IL-2-induced VLS therapy, suggesting that adaptive immunity was not required. In contrast, studies with WT mice and humanized mice (irradiated and reconstituted with human CD34^+^ stem cells) have indicated that T regulatory cells (Tregs) negatively regulate IL-2-induced VLS by suppressing proinflammatory cytokine and chemokine levels [22, 23]. In addition, Treg depletion by anti-CD25 or anti-CTLA4 antibodies increases the severity of VLS in mice [22]. However, CD25 and CTLA4 are expressed on other cell types and the observed result might be only in part due to the depletion of Tregs. Additional studies utilizing FoxP3 conditional knockout mice and adoptive cell transfer of Treg cells will be necessary to further elucidate the role of CD4^+^ Tregs in IL-2-induced VLS. Taken together, decades of research tell us that IL-2-induced VLS has a complex etiology, rendering it difficult to translate our understanding from mouse to human.

Furthermore, it is understood that IL-2 has a critical role in regulating immunologic tolerance by supporting the development and maintenance of FoxP3^+^ Tregs [2]. Tregs are unable to produce IL-2 due to FoxP3-driven transcriptional repression of the IL-2 locus and, as such, are dependent on the production of IL-2 from other immune cells to survive and function in the periphery [2, 24]. Tregs constitutively express high levels of IL-2R*α*, and thus IL-2R*αβγ*, and readily compete with other IL-2R-bearing cells for IL-2 [2, 25]. It can be expected that IL-2 therapy expands Treg cell populations due to the high baseline expression of IL-2R*αβγ*. Analysis of the CD4^+^ T cell compartment in cancer patients showed expansion of CD25^+^ FoxP3^+^ Tregs following IL-2 therapy [26]. High Treg infiltration into tumors has been correlated with a worse disease prognosis and therefore implicates Tregs as a detrimental factor for cancer patients [9].

## 3. Current IL-2-Based Fusion Proteins for Cancer Therapy

This section discusses existing IL-2-based strategies for cancer immunotherapy that have emerged in an attempt to circumvent the shortcomings and improve efficacy compared to the original high-dose strategy. Chimeric IL-2 constructs are generated by genetic fusion of DNA sequences of IL-2 and a protein of interest. PEG-IL2 is formed through chemical reactions that fuse polyethylene glycol (PEG) molecules to IL-2. Although IL-2 is utilized in a wide variety of settings, we have chosen to specifically discuss IL-2-based fusion proteins used in a cancer therapeutic setting. Select IL-2-based fusions currently in preclinical/clinical development are highlighted in Table 1.

### 3.1. Albumin

IL-2 has a very short circulating half-life of approximately 7 minutes in humans and is rapidly sequestered and removed by the kidneys [27, 28]. Genetic fusion to the serum protein albumin presents an appealing strategy to augment the pharmacokinetic properties of IL-2. The half-life of albumin is approximately 3 weeks due to a pH-sensitive, neonatal Fc receptor- (FcRn-) mediated recycling mechanism that rescues albumin from lysosomal degradation pathways [29–32]. Furthermore, albumin is a ubiquitous protein and cycles through blood and lymphatics [33]. As such, albumin can deliver protein payloads to key immunological tissues such as lymph nodes and the spleen. CTLL2 cells are an IL-2-dependent T cell line and are considered the gold standard for assaying IL-2 [34]. Yao et al. described a fusion between albumin and IL-2 (Alb-IL2) that was able to maintain CTLL2 cell activation and induction of proinflammatory cytokines *in vitro*, albeit slightly less potently than when compared to recombinant IL-2 (rIL-2) [35]. *In vivo* pharmacokinetics and biodistribution analysis revealed that Alb-IL2 preferentially accumulated in the liver, lymph nodes, and spleen of mice, whereas IL-2 was cleared rapidly by the kidneys. IL-2 was cleared approximately 50 times faster, supporting the notion that fusion to albumin can dramatically increase the bioavailability of IL-2 [36].

It is important to address whether improved pharmacokinetic properties translate to increased anti-tumor immune responses. In murine tumor models, Alb-IL2 had superior tumor control and decreased liver metastasis, leading to improved overall survival. Immunohistochemistry performed on murine tumor sections revealed strong staining of tumor-associated CD3^+^ T cells, primarily consisting of CD8^+^ T cells, indicating that Alb-IL2 could induce effective adaptive immune responses [36]. Together, these preclinical studies of Alb-IL2 suggest that this strategy can increase circulating half-life, further potentiating the potent biological effects of IL-2. In patients, this may translate to better efficacy as well as more convenient dosing schedules that will ultimately improve patient quality of life. In that regard, two phase I trials have been initiated for Alb-IL2 (Albuleukin); however, the current status of clinical testing is unknown [37] (Table 1).

### 3.2. Fragment Crystallizable (F_C_)

A key property of the antibody IgG subclass is the prolonged serum half-life of approximately three weeks in humans. Similar to albumin, a pH-dependent FcRn recycling mechanism circumvents lysosomal degradation and allows antibodies to remain in circulation longer than other biological molecules [38]. Genetic fusion of an Fc region to a protein of interest improves pharmacokinetics in an antigen-independent manner and has led to FDA approval of numerous constructs for the treatment of human diseases [39, 40]. Specifically, IL2-Fc has been utilized in preclinical models to enhance the magnitude and duration of adaptive and innate immune responses that occur in viral infections and cancer. IL2-Fc has been tested in combination with antiretroviral drugs in rhesus monkeys infected with simian immunodeficiency virus [41]. Moreover, the extended IL-2 half-life provided by Fc fusion potentiates the innate and adaptive antitumor response. For example, the combination of a tumor-targeted antibody with IL2-Fc (bearing D265A mutation to abolish Fc effector function) treatment had superior tumor control, mediated by CD8^+^T cells, NK cells, macrophages, and polymorphonuclear cells [42].

Recently, two novel IL-2 fusion molecules utilizing Fc components in combination with other moieties have emerged. A construct termed “pro-IL2” contains superkine IL-2 (sumIL-2) [43] fused with the extracellular domain of the IL-2R*β* and a WT Fc domain. The IL-2R*β* portion is attached via a cleavable linker, allowing IL-2 activity to be restricted until cleavage occurs in the tumor microenvironment [44]. Preclinical analysis demonstrated that pro-IL2 had comparable anti-tumor efficacy to sumIL2-Fc with a superior toxicity profile. Another novel construct, CUE-101, is aimed at expanding HVP16 E7_11-20_-specific CD8 T cells for the treatment of HPV+ cancers. CUE-101 contains 2 E7-peptide-HLA complexes, 4 reduced affinity IL-2 molecules, and an IgG1 Fc domain. CUE-101 can bind, activate and expand E7-specific CD8 T cells which translated to anti-tumor efficacy and formation of immunological memory [45]. CUE-101 is currently undergoing a phase I clinical trial (NCT03978689) [46].

In addition to altered pharmacokinetics, the Fc component can provide additional functions. The Fc domain contains binding sites for Fc*γ*R and C1q, leading to antibody-dependent cellular cytotoxicity (ADCC) and activation of the complement cascade, respectively. Vazquez-Lombardi and colleagues explored two different IL2-Fc constructs with intact Fc effector functions: WT IL2-Fc and a triple IL-2 mutant version (R38D, K43E, E61R; IL2^3x^-Fc) that abolished binding to CD25 [47]. Although IL2^3x^-Fc was able to increase CD8^+^ and NK cell populations more so than the WT version, WT IL2-Fc resulted in superior tumor control. Further study revealed that the antitumor effect was achieved by Fc-mediated ADCC of Treg cells. Tregs express high levels of the high-affinity IL-2R and readily bind IL-2 and thus IL2-Fc, in the environment. It has become apparent that Tregs infiltrating the TME leads to a poor prognosis [9]. Fusion of IL-2 to an Fc domain is a promising strategy to improve the pharmacokinetic profile of IL-2, selectively deplete Tregs, and increase antitumor immune responses. Clinical trials will be necessary to explore the utility of IL2-Fc for the treatment of cancer.

### 3.3. Antibody Targeted (Immunocytokines)

In contrast to Fc-based fusion proteins, immunocytokines are the fusion of an antigen-binding domain of an antibody and a protein payload, such as a cytokine [48]. The immunocytokine format improves the biodistribution profile through antigen-dependent targeting. For example, IL-2 that is released locally from activated cells or administered exogenously requires high doses to ensure that IL-2 reaches its intended targets in relevant tissues. To overcome this issue, immunocytokines can be employed to deliver IL-2 to a tissue of interest, thereby reducing systemic inflammation and the need for high-dose regimens. Immunocytokines can utilize all, or part of, an antibody molecule and typically have the protein payload attached at the C terminal end to leave the antigen-binding domain unobstructed. Immunocytokines can be engineered in various ways to alter molecular weight, avidity, and half-life. Careful consideration must be taken when determining the target for immunocytokines. The target antigen must be highly expressed in tumor tissue and low or no expression in healthy tissue. Expression of these antigens elsewhere can sequester the immunocytokine from its intended location and provoke unwanted inflammation and adverse side effects.

Immunocytokines containing IL-2 are among the most developed [49–51]. As a consequence of rapid growth and competition for nutrients, tumors differentially express several potential antigens (i.e., growth factor receptors) that serve as targets for IL-2 immunocytokines. Furthermore, the continually evolving vascular network in tumor tissue results in tumor localized expression of various splice forms of extracellular matrix proteins. IL-2 Immunocytokines have been generated that target extracellular matrix proteins of the tumor vasculature such as the splice isoforms of fibronectin (extra domains A and B) [52–57] and A1 domain of Tenascin C [58–60] or to overexpressed surface antigens including carcinoembryonic antigen (CEA) [61], epithelial cell adhesion molecule (EpCAM) [62], programmed death-ligand 1 (PD-L1) [63, 64], epidermal growth factor receptor (EGFR) [65], disialoganglioside (GD2) [62, 66, 67], and CD20 [68]. Additionally, unconventional antigens have been targeted, such as phosphatidylserine [69] and nuclear antigens [70]. The IL-2 immunocytokines currently in preclinical and clinical development are reviewed in detail here [49–51].

### 3.4. Immunotoxins (Diphtheria)

Immunotoxins, which are a fusion between an immunological agent (i.e., cytokine) and a toxin, have been a longstanding approach for targeted cytotoxicity. Researchers in the 1980s developed DAB489-IL2, which consisted of IL-2 fused to diphtheria toxin (DT) at the DT receptor binding site. A second-generation molecule, DAB389-IL2 (Denileukin Diftitox, Ontac), with a 97-amino acid in-frame deletion led to a construct with greater affinity for its target, enhanced potency, and extended half-life [71, 72]. Denileukin Diftitox is targeted to cells bearing the intermediate or high-affinity IL-2 receptor. Once the fusion protein engages the IL-2R, the receptor-ligand complex is internalized. Inside the endosome, the toxic fragment of DT, fragment A, can escape into the cytoplasm and interfere with protein synthesis to initiate the death of the target cell [73].

Denileukin Diftitox has been widely used for cutaneous T cell lymphoma (CTCL) because malignant lymphocytes have high expression of the IL-2R. Although Denileukin Diftitox demonstrated modest success in clinical testing, it ultimately received FDA approval for persistent or relapsed CTCL in 1999 [74, 75]. Clinical trial results indicate that patients still suffer from IL-2-related toxicities such as fever, flu-like symptoms, and VLS in some cases. A single point mutation, V6A, introduced into the molecule greatly improved the toxicity profile and reduced VLS compared to the WT molecule [76]. Additional studies and clinical trials will be necessary to determine if this modified construct has improved safety and efficacy in humans.

It is reasonable to consider that Denileukin Diftitox affects nonmalignant IL-2R^+^ lymphocytes that are necessary to help fight cancer spread. Reports have suggested that Tregs are preferentially depleted compared to effector cells [77, 78]. Elimination of Treg cells disrupts the balance of activation and suppression to allow for expansion of tumor-specific clones [77]. Additional research is needed to understand how Denileukin Diftitox affects various immune and nonimmune IL-2R expressing cells and whether that supports or weakens antitumor responses.

### 3.5. Polyethylene Glycol

The idea of conjugating PEG chains to IL-2 for extended circulating half-life originated in 1987, launching nearly a decade of research and clinical trials exploring PEG-IL2 for cancer immunotherapy [79]. The original report showed that PEG-IL2 maintained its biological function and was more potent at controlling tumor growth compared to IL-2 [79]. L10 tumor-bearing mice (guinea pig hepatocarcinoma L10 cell line) treated with PEG-IL2 showed reduced tumor growth compared to phosphate-buffered saline-treated controls [80]. Pretreatment with antithymocyte antibodies abrogated the antitumor effects, signifying that PEG-IL2 was able to stimulate T cells to kill the tumor [80]. Mice were able to reject a tumor rechallenge, suggesting PEG-IL2 induced strong systemic immunity. The observed antitumor effect was mediated primarily by T helper subsets, and cytotoxic T cells played a minimal role [81]. In vitro and preclinical data supported the initiation of phase 1 clinical testing [82, 83]. However, clinical testing utilizing high-dose (HD) IL-2 along with PEG-IL2 revealed no therapeutic benefit, resulting in the termination of PEG-IL2 research.

Nearly two decades after PEG-IL2 research was terminated, Nektar Therapeutics published a report describing a novel PEG-conjugated IL-2 molecule, NKTR-214 (Bempegaldesleukin) [84]. In contrast to the original formulations that had many stochastically bound PEG chains, NKTR-214 is engineered to have ~6 releasable PEG chains located on lysine resides near the CD25 binding domain [84, 85]. The location of the PEG chains generates an IL-2R-biased agonist by diminishing the ability of IL-2 to bind to IL-2R*α* while leaving interactions with IL-2R*β* and IL-2R*γ* intact, allowing for selective targeting of CD8^+^ T cells and NK cells over Tregs. Moreover, NKTR-214 is administered as an inactive prodrug, requiring hydrolysis of several PEG chains to generate the active forms, 2-PEG-IL2 and 1-PEG-IL2 [85]. The prodrug strategy limits the overwhelming systemic toxicities that are associated with the infusion of HD IL-2 and maintains the extended circulating half-life. Preclinical testing showed that NKTR-214 potently increased the CD8^+^:Treg ratio in tumors compared to rIL-2 (Aldesleukin), translating to better tumor control. Safety and toxicity studies in monkeys revealed an acceptable safety profile [84]. Moreover, NKTR-214 outperformed IL-2 in supporting T cell activation and persistence in an adoptive cell therapy model [86].

Preclinical data demonstrating improved pharmacokinetics, immune activation, and reduced toxicities warranted the initiation of phase 1 clinical testing. A phase 1, first-in-human trial was initiated for patients with advanced solid tumors who have failed one or more other treatment options [87]. Delivered as a monotherapy, 21.4% of patients observed some degree of tumor reduction and 53.8% of patients had stable disease. Strong immunologic changes were observed in the blood and tumor microenvironment (TME), marked by increases in CD8^+^ and CD4^+^ T cells and NK cells. Although the expansion of Tregs was observed in the periphery, little expansion of Tregs was seen in the tumor milieu. Cells displayed markers of activation (ICOS, CTLA4, PD1, OX40) and transcriptional analysis indicated upregulation of genes associated with immune activation and effector function. Mechanistic studies in mice revealed that decreased Treg frequency in tumors was due to effector CD8^+^ T cells secreting high levels of IFN*γ* and TNF*α*, acting locally on Tregs to decrease proliferation and increase apoptosis [88]. This specificity for the tumor and not the periphery is likely due to CD8^+^T cells encountering their cognate antigen in the TME. Currently, NKTR-214 is being evaluated in several phase I-III clinical trials (see clinicaltrials.gov).

Recently, Sanofi has developed a PEG-IL2 fusion, THOR-707 (SAR444245), that contains one permanent PEG chain located on a novel amino acid insertion. The location of the PEG chain blocks the ability of IL-2 to bind to IL-2R*α*, generating a molecule biased towards CD8 T cells and NK cells expressing the IL-2R*βγ* [89]. THOR-707 is currently undergoing phase I testing in patients [46]. Taken together, PEG fusions have delivered promising results, and more research is needed to determine if this strategy ultimately improves efficacy and patient tolerability over conventional IL-2 therapy.

### 3.6. NKG2D

A great amount of focus has been on improving survival, proliferation, and effector function of CD4^+^ and CD8^+^ T cells, but other immune cells such as NK cells also readily respond to IL-2 and are important for mediating cytotoxicity towards tumor cells. NK cells express high levels of the surface protein, NKG2D, which acts as an activating receptor for the NK cell when it engages cognate ligands on stressed cells [90]. Orthopoxvirus MHC-I-like protein (OCMP) is known to bind NKG2D with high affinity [91, 92]. An OCMP-mutIL-2 fusion protein targets cells bearing NKG2D and contains mutations that bias IL-2 towards binding the *βγ* chains of the IL-2R. It was found that OCMP-mutIL2 causes robust activation and proliferation of NK cells in vitro and in vivo, relative to WT or mutant IL-2 alone. Mice treated systemically with OCMP-mutIL-2 had improved tumor killing and overall survival as a result of NK cell expansion [93].

NKG2D can bind several stress-induced ligands on cells, including MIC A/B and Rae-1. Expression of these ligands signals indicates that a particular cell is stressed, malignant or infected, and it needs to be removed from the tissue [90]. The TME can present many environmental challenges to cells, leading to an upregulation of NKG2D ligands in the tumor milieu [94]. Fusion proteins utilizing NKG2D can preferentially target those stress-induced ligands and accumulate in the TME. An NKG2D-Fc-IL2 DNA vaccine in combination with a therapeutic human papillomavirus type 16/E7 peptide vaccine was able to result in accumulation and proliferation of E7-specific T cells at the tumor site and reduced tumor growth that resulted in prolonged survival of tumor-bearing mice [95]. Together these reports suggest that targeting additional IL-2R bearing cells may be a promising strategy to improve IL-2 based therapies.

## 4. Concluding Remarks and Future Directions

**Figure 1:**
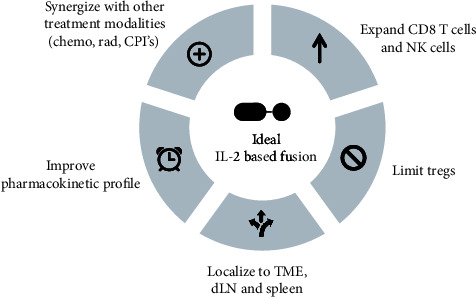
Properties of a next-generation IL-2-based fusion construct. A next generation IL-2-based fusion protein for cancer immunotherapy will encompass the criteria shown to overcome challenges associated with conventional IL-2 therapy. Due to the pleiotropic effects of IL-2 on different IL-2R-bearing immune cells, a biased agonist targeting CD8 T cells and NK cells bearing the intermediate affinity receptor, IL-2R*βγ*, will promote antitumor immunity and limit expansion of immunosuppressive Tregs. Furthermore, traditional IL-2 therapy is limited by a short half-life, poor *in vivo* localization, and off-target activity which all contribute to adverse effects experienced by patients. Strategies that improve the pharmacokinetic and biodistribution profile of IL-2 will increase antitumor efficacy. Lastly, IL-2-based therapy delivered in combination with other treatment modalities will provide the best opportunity to overcome immunosuppression and immune escape that occurs. Tregs: T regulatory cells; TME: tumor microenvironment; dLN: draining lymph node; chemo: chemotherapy; rad: radiation; CPI: checkpoint inhibitor.

IL-2 is a potent cytokine capable of regulating adaptive immune responses that are critical for antitumor immunity. Through the genetic or chemical attachment of additional protein domains, chimeric IL-2 fusion proteins have emerged as promising alternatives to traditional IL-2 therapy. Although the chimeric fusion proteins outlined in this article have provided some relief to the drawbacks of HD IL-2, there is still opportunity to improve these strategies. Based on our current understanding of IL-2 biology and recent advancements, we have proposed several criteria that are critical components of next-generation IL-2 fusion proteins (Figure 1).

It is well understood that IL-2 is a pleiotropic cytokine positioned at the crossroads of T cell activation and regulation. As such, it is imperative to engineer an IL-2 molecule that is biased towards effector cells bearing the intermediate affinity receptor, such as naïve and memory CD8^+^ T cells and NK cells, versus Treg cells that express the high-affinity receptor. Reducing the immunosuppressive barrier will be critical for generating durable antitumor immune responses. Additionally, the biodistribution profile of IL-2 must be improved considering that some nonimmune cells express low to intermediate levels of the trimeric IL-2R [19]. Strategies that preferentially deliver IL-2 to the draining lymph nodes, spleen, and tumors will position IL-2 near effector T cells and NK cells. Directing IL-2 towards these cell types will reduce the severity and occurrences of IL-2-induced VLS and other related toxicities that are a major concern for patients. Relatedly, having an IL-2 molecule with enhanced pharmacokinetics will ensure effective penetration into these target tissues. Less frequent dosing as a result of an improved half-life will alleviate unnecessary systemic inflammation and allow the drug to be conveniently administered in the outpatient setting.

Lastly, multifaceted treatment regimens are likely to achieve the best objective response rates because of cancer's complex etiology [96]. In addition to circumventing toxicities and side effects, IL-2-based therapies must be able to synergize with standard-of-care treatments including surgical resection, radiation therapy, and chemotherapy. Several of the immunocytokine constructs listed in Table 1 are currently being tested in conjunction with radiotherapy or chemotherapy [97–100]. Furthermore, it is important to consider combining therapies that nonredundantly target immune activation and suppression pathways. Immune checkpoint inhibitor (CPI) therapies, including anti-PD(L)1 and anti-CTLA4, have been launched to the forefront of cancer treatment in the last decade. CPIs may be ideal for combination treatments because cytokine therapies support proliferation and differentiation of effector cells, while CPIs block the regulatory pathways that attenuate an immune response [101]. For example, Bempegaldesleukin is currently in clinical testing with Atezolizumab, Nivolumab, and Ipilimumab [14, 102]. Achieving the balance between clinical efficacy and low toxicity will remain a challenge, particularly when a patient is receiving multiple treatment types.

In conclusion, IL-2 has been a cornerstone of cancer immunotherapy because of its critical role in the T cell activation pathway. Despite having FDA approval for RCC and metastatic melanoma, poor response rates and adverse side effects have curtailed the widespread adoption of HD IL-2 therapy. The development of an IL-2-based therapy that can preferentially expand CD8 T cells and NK cells limits adverse side effects and improves clinical efficacy will potentially be a powerful tool in the cancer treatment paradigm.

## Figures and Tables

**Table 1:** Select IL-2-based fusion constructs for cancer immunotherapy.

Fusion strategy		Description	Indication	Clinical status	Reference
Albumin	Albuleukin	Human serum albumin fused to IL-2	Solid tumors	Phase I	[37]
Fc	IL2-fc/IL2-Fc^3x^	Murine IgG2c Fc region fused to IL-2. Triple mutant version contains R38D, K43E, and E61R mutations to attenuate Fc effector functions	B16F10 and CT26 tumor models	Preclinical	[47]
	CUE-101	IgG1 Fc with attenuated effector function, 2 peptide-HLA complexes, and 4 reduced affinity IL-2 domains	HPV+HNSCC	Phase I	[45]
	ProIL-2/sumIL2-fc	Human IgG1 Fc region fused to “superkine” IL-2 and a cleavable IL-2R*β* domain	MC38 and B16 tumor models	Preclinical	[44]
Immunocytokine	L19-IL2	L19 antibody is specific for EDB splice isoform of fibronectin	Metastatic melanoma and renal cell carcinoma	Phase I/II	[97, 103, 104]
	F8-IL2	F8 antibody is specific for EDA splice isoform of fibronectin	Metastatic NSCLC and CT26 mouse models	Preclinical	[49, 55, 56, 105]
	F16-IL2	F16 antibody specific for A1 domain of tenascin C	Glioblastoma, breast cancer	Preclinical, phase I/II	[58, 60, 98]
	hu14.18-IL2	hu14.18 antibody is specific for disialoganglioside (GD2)	Neuroblastoma, metastatic melanoma	Phase I/II, phase II	[106–108]
	huKS-IL2 (EMD 273066, tucotuzumab celmoleukin)	huKS antibody is specific for epithelial cell adhesion molecule (EpCAM)	EpCAM+advanced solid tumors	Phase I	[99]
	NHS-IL2 LT (EMD 521873, selectikine)	NHS antibody is specific for necrotic DNA fused to IL-2 containing a D20T mutation	Advanced solid tumors, metastatic NSCLC	Phase I	[100, 109]
	B3-IL2	Heavy chain only antibody fragment specific for PD-L1	Pan02 and KPC tumor models	Preclinical	[63]
	2aG4-IL2	2aG4 antibody is specific for exposed phosphatidylserine on cell surface	4T1 mouse model	Preclinical	[69]
Immunotoxin	DAB389-IL2 (Ontak)	DT with a 97 amino acid in-frame deletion fused to IL-2 at the DT receptor binding site	CTCL, CLL, non-Hodgkin's lymphoma, NSCLC	FDA approved for CTCL	[75]
PEG	Bempegaldesleukin (NKTR-214)	IL-2 with approximately 6 cleavage PEG chains positioned near the CD25 binding domain	Advanced or metastatic solid tumors	Phase I/II	[86, 87]
	THOR-707 (SAR444245)	IL-2 with one noncleavable PEG chain attached to a novel amino acid insertion	Solid tumors	Preclinical, phase I/II	[46, 89], NCT04009681
